# Effect of zinc cations on the kinetics of supramolecular assembly and the chirality of porphyrin J-aggregates†
†Electronic supplementary information (ESI) available: UV/Vis spectral changes during metallation (SI1) and demetallation (SI2) of TPPS, RLS profiles (SI3), fluorescence emission decay and time resolved and fluorescence anisotropy (SI4), *m* and *n* kinetic parameters as a function of Zn(ii) (SI5), ICP-OES analysis experimental conditions (Table SI1), and acquisition parameters for ICP-OES analysis (Table SI2). See DOI: 10.1039/c6sc02686a



**DOI:** 10.1039/c6sc02686a

**Published:** 2016-09-29

**Authors:** A. Romeo, M. A. Castriciano, R. Zagami, G. Pollicino, L. Monsù Scolaro, R. F. Pasternack

**Affiliations:** a Istituto per lo Studio dei Materiali Nanostrutturati ISMN-CNR c/o Dipartimento di Scienze Chimiche, Biologiche, Farmaceutiche ed Ambientali , University of Messina , V.le F. Stagno D'Alcontres, 31 , 98166 Messina , Italy . Email: castriciano@pa.ismn.cnr.it; b Dipartimento di Scienze Chimiche, Biologiche, Farmaceutiche ed Ambientali , University of Messina , C.I.R.C.M.S.B , V.le F. Stagno D'Alcontres, 31 , 98166 Messina , Italy; c Dipartimento di Scienze Biomediche, Odontoiatriche e delle Immagini Morfologiche e Funzionali , Sezione SASTAS , University of Messina , Messina , Italy; d Department of Chemistry and Biochemistry , Swarthmore College , Swarthmore , Pennsylvania PA 19081 , USA

## Abstract

Dilute aqueous solutions of anionic *meso*-4-sulfonatophenyl-porphyrin (TPPS) extract zinc(ii) ions from glass or quartz surfaces at room temperature and efficiently form the corresponding metal complex (ZnTPPS). The partial or complete formation of ZnTPPS has been probed by UV/Vis spectroscopy and both static and time-resolved fluorescence. The source of zinc(ii) ions has been clearly identified through inductively coupled plasma optical emission spectrometry. The presence of increasing amounts of ZnTPPS slows down the rate of TPPS J-aggregate formation in acid solution. This influences the nucleation step and has a profound impact on the onset of chirality in these species. This evidence indicates the important role of this adventitious metal ion in the interpretation of various spectroscopic and kinetic data for the self-assembly of the TPPS porphyrin and provides some insights into controversial findings on their chirality. The use of this metal derivative as the starting compound for *in situ* formation of monomeric TPPS is suggested.

## Abstract 2

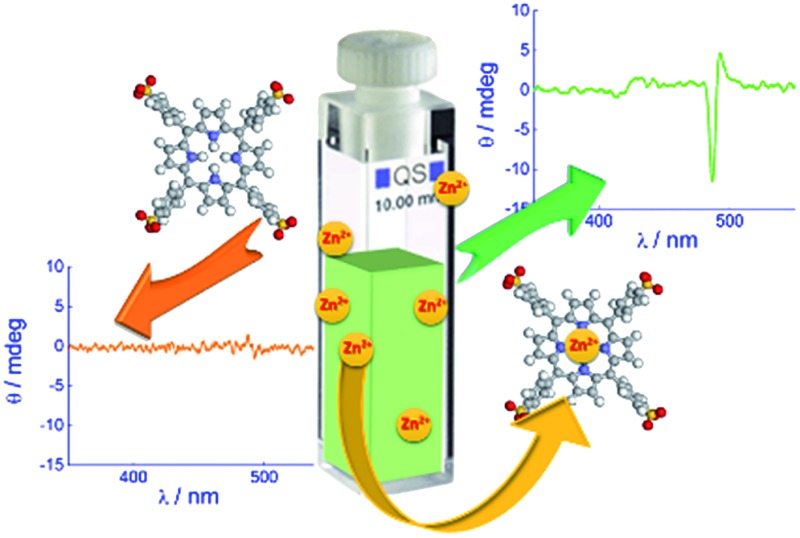
The key role of adventitious zinc(ii) ions, extracted from glass and quartz surfaces, in the kinetics of porphyrin aggregation and in the subsequent expression of their chirality is discussed herein.

## Introduction

Putative “spontaneous” induction of optical activity in supramolecular assemblies of achiral entities in the absence of chiral templates is a subject of considerable and growing interest.^1^ An understanding of this effect paves the way to proposals for “natural” mirror-symmetry breaking, which is ubiquitous in the universe.^2^ Porphyrin derivatives – such as *meso*-4-sulfonatophenyl-porphyrin (TPPS) – that have been shown to self-assemble into chiral supramolecular structures are especially attractive for such investigations due to the ease with which they can be tuned and to their rich spectral properties.^3–7^ The hierarchical self-assembly processes show different thermodynamically and kinetically controlled paths related to porphyrin concentration and medium conditions such as pH and ionic strength.^3,8,9^ The aggregation is driven by electrostatic (including hydrogen bonding) and hydrophobic interactions occurring between the adjacent porphyrins, and lead to arrays whose sizes span from nano- to microscale.

Previously we have shown that when optically active tartrate is employed to induce aggregation of TPPS, Cotton effects of different sign are obtained for the chiral assemblies, raising the intriguing issue of transmission of chirality from a local or molecular level to the mesoscopic regime.^4,9^ Symmetry breaking has also been reported to occur through the stirring of porphyrin solutions during their aggregation, with a statistical correlation between the stirring sense and the CD signal observed in the aggregate absorption regions.^10^ In a recent report, the handedness of chiral porphyrin J-aggregates has been controlled by applying rotational, gravitational and orienting forces at the beginning of the assembly process, underscoring the importance of the nucleation step on the product aggregates.^11,12^ Yet, it must be noted that mechanical swirling as well as adding chiral templating reagents constitute external driving forces to promote and control chirality induction.

A controversial topic deals with the unpredictable optical activity observed for TPPS J-aggregates obtained in aqueous solutions under varying experimental conditions in the absence of any added chiral templating agent or directional agitation.^13,14^ One proposal is that this symmetry breaking is due to the adventitious presence of traces of chiral impurities,^15,16^ *i.e.*, it is once again due to an external driving force. In our laboratories, we are currently exploring other possibilities for the spontaneous appearance of chirality.

Recent reports have described intrinsic chiral arrangements of monomeric units into a sheet-like architecture as determined by X-ray^15,16^ or into helical nanotubes as probed by electron diffraction methods.^17^ The apparent symmetry-breaking of TPPS J-aggregate helical tubes has been proposed as being due to small chiral aggregates such as dimers, tetramers, and other small oligomers which are present in the starting solution. Other evidence for the role of small aggregates (“seeds”) comes from studies of the kinetics of assembly formation. When acid is added to dilute TPPS solutions (at micromolar concentrations), the formation of J-aggregates follows a sigmoidal profile that is characterized by a rate-determining-step involving the formation of a “critical size” assembly which catalyzes further growth.^13^ The dimerization or oligomerization process, which precedes aggregation into much larger clusters, is exothermic for porphyrins and other aromatic compounds,^18–21^ thus suggesting the possibility of removing such aggregate precursors from solution by heating.

The crucial role of the reagent mixing protocol on kinetic rates and aggregate size has been explored.^3,9,13,22^ These findings point once again to the importance of the preformed “seeds” in the reactant solution that profoundly affect the kinetics and consequently the chirality of the aggregates. The presence and importance of such species provide an explanation for apparent discrepancies and the apparent lack of reproducibility in the literature; one difficulty in obtaining reproducible results is that the number and size distribution of “seeds” vary from porphyrin sample to sample.

In this report, we (i) describe several observations on the reactivity of TPPS that might well account for some of the inconsistencies found in the literature and (ii) propose an experimental strategy that appears to circumvent these difficulties. We will show that TPPS is able to efficiently extract and bind zinc(ii) ions from the surface of glass or quartz when in contact with its neutral solutions. The presence of this metal complex – always present in varying quantities depending on the experimental conditions – affects the kinetics of protonation of the central core, influencing the chirality of the aggregates.

## Results and discussion

**Fig. 1:**
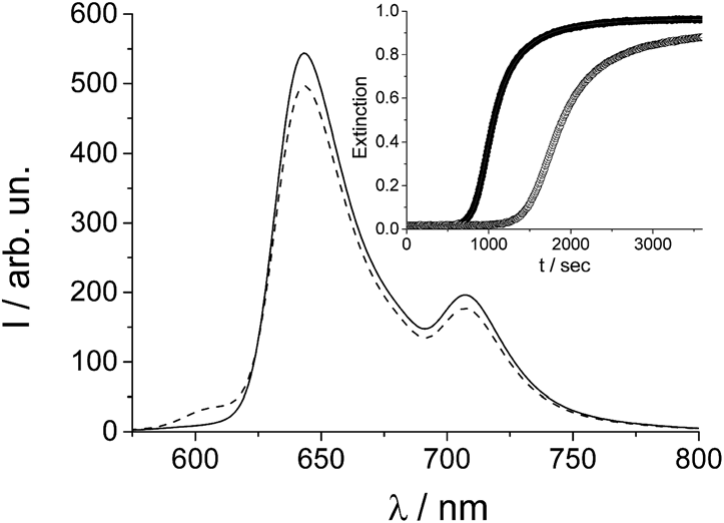
Fluorescence emission spectra of 2 μM TPPS solution at room temperature (full line), and after thermal treatment (dashed line). In the inset the kinetic profiles at 491 nm for TPPS aggregation induced by HCl in untreated (solid circles), and in treated solution (empty circles) are shown; [TPPS] = 3 μM, [HCl] = 0.5 M, PF protocol; *T* = 298 K. The solid lines represent the best-fitting of the experimental data (*λ*_491 nm_) to eqn (1): (*k*_0_ = (1.4 ± 0.1) × 10^–5^ s^–1^; *k*_c_ = (1.25 ± 0.01) × 10^–3^ s^–1^; *m* = 4.3 ± 0.04; *n* = 12 ± 0.1; *R* ^2^ = 0.99995) (full circles), (*k*_0_ = (6.7 ± 0.1) × 10^–6^ s^–1^; *k*_c_ = (6.9 ± 0.02) × 10^–4^ s^–1^; *m* = 4.2 ± 0.06; *n* = 12 ± 0.1; *R* ^2^ = 0.99996) (empty circles).

To verify the presence and role of preformed nuclei in the aggregation processes and on the chirality of the supramolecular systems, we performed a series of kinetic experiments on samples subjected to different thermal treatments. “Untreated” samples (from freshly made stock solutions) have been taken as a reference. “Treated” samples involved solutions of TPPS porphyrin at micromolar concentrations, which have been gradually heated up to 333 K (at a rate of 2 K min^–1^), maintained at this temperature for 30 min and then cooled down to 298 K before aggregation. When HCl solution (up to 0.5 M) is added to a micromolar solution of TPPS, aggregation is fostered (porphyrin first or PF protocol). One such set of kinetic traces for 3 μM TPPS solutions obtained from the extinction at 491 nm (corresponding to the J-aggregate extinction band) is reported as the inset of Fig. 1. The time evolution of the trace corresponding to the reference sample (filled circle points) and treated sample (empty circle points) both display sigmoidal profiles characterized by almost flat initial lag periods corresponding to early stage nucleation (much longer for the treated sample) followed by quite rapid growth of the aggregates. Analysis of the kinetics was performed using a previously reported autocatalytic pathway model.^23,24^ Apart from the different initial lag times, the traces at 491 nm reveal that the value for the observed rate constant *k*_c_, corresponding to the untreated sample, is about 60% higher than that observed for the thermally treated one. In order to clarify the basis for the observed difference in rates, we performed further spectroscopic investigations on the starting porphyrin solutions. Since fluorescence emission spectroscopy is an especially sensitive optical detection technique, we used this method to verify the stability of the samples after thermal annealing and to detect the possible presence of other species in solution.

The fluorescence emission spectrum, shown in Fig. 1, measured on the treated porphyrin sample displays two emission bands at 644 and 708 nm typical for TPPS porphyrin plus an additional weak band centered at 606 nm (dashed trace) which is completely absent in the untreated sample (full line).

In order to determine the nature of this new species, we collected UV/Vis and fluorescence emission spectra on about 50 samples of dilute porphyrin solution at micromolar concentrations (1–10 μM) in glass vials at different temperatures (333–373 K) for different times (30 min to 3 days).

**Fig. 2:**
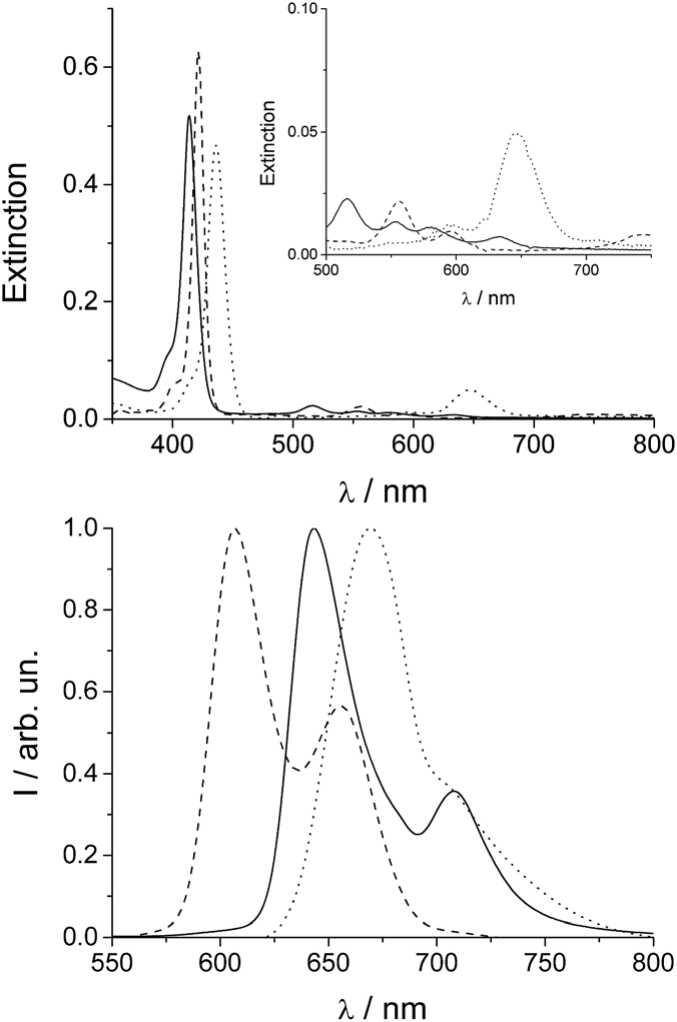
UV/Vis (upper plot) and normalized fluorescence emission spectra (lower plot) of freshly made aqueous solutions of TPPS (solid line), after thermal annealing (dashed line) and soon after acidification of the thermally annealed solution ([HCl] = 0.5 M, dotted line). The inset of the upper plot shows an expansion of the Q-band region. [TPPS] = 1 μM, *T* = 298 K.

What is observed is almost total conversion in the cases of the most dilute samples (1 μM) of the free base porphyrin to this new species (see the ESI, Fig. SI1†) and only partial and random conversion independent of temperature and time for all the other samples. The UV/Vis spectra and the corresponding fluorescence emission spectra for a 1 μM sample are reported in Fig. 2 (dashed traces).

TPPS is readily soluble in aqueous solution, displaying a very sharp and intense B-band centered at 414 nm, accompanied by four weaker Q bands (516, 554, 580, and 634 nm) in the absorption spectra (Fig. 2, solid line). Experimental evidence suggests that anionic TPPS at micromolar concentrations in polar solvents containing near zero ionic strength is present as a monomer and its spectroscopic behaviour is not affected by aging. In contrast, it has been reported that in solutions of short chain alcohols of differing polarity^25^ as well as in the AOT microemulsion water pool^26^ the free base porphyrin is converted to a new species with a B-band centred at 423 nm, which is ascribed to a J-type dimer of the neutral porphyrin or H-type dimer of the diacid porphyrin. In the present case, the UV/Vis spectra display the presence of a band centred at 422 nm together with two Q-bands at 556 and 598 nm (Fig. 2, upper panel, dashed lines). The time for the development of this new band, bathochromically shifted with respect to the B-band of the free base porphyrin, is variable from a couple of hours to one month, depending on the conditions. The observed final spectra do not further evolve with time. The presence of two Q-bands points to an increased symmetry of the porphyrin core. The emission spectrum of the final species (Fig. 2, lower panel) shows two bands (606 and 654 nm) blue-shifted with respect to that of the initial porphyrin aqueous solution. A time resolved fluorescence emission experiment has been performed on the species obtained after thermal annealing. The fluorescence emission decay shows a mono-exponential behaviour with a lifetime value of 1.9 ns (Fig. SI4†). Resonance light scattering (RLS) is consistent with a monomeric or small aggregate form of this species. Typical RLS profiles for these solutions are characterized by a “well” corresponding to the position of the B-band in the UV/Vis spectra arising from photon absorption (see the ESI Fig. SI3†).^27^ All the spectroscopic features observed for this “unknown” species enable us to unequivocally identify it as the zinc(ii) metal complex of TPPS. Zinc(ii) is known to be a quite labile metal ion and, under acidic conditions, demetallation of the zinc porphyrin occurs at a rate dependent on pH (see the ESI Fig. SI2†).^28^ Acidification of these solutions with pH < 1 shows almost instantaneous formation of the diacid species as suggested by the UV/Vis and fluorescence emission spectral features (Fig. 2, dotted lines). Therefore, we conclude that after thermal annealing of the porphyrin solutions the Zn(ii) derivative forms, even if we exclude the presence of trace metal contamination in the solution. Since, as has been reported, TPPS is able to interact with hydrophilic surfaces, *e.g.* adsorption on fused quartz forming a monolayer of the free-base porphyrin,^29^ or to form metallo-derivatives, we set about determining whether trace zinc contamination of the solutions arises due to the glass/quartz surface. Consequently, we used inductively coupled plasma optical emission spectrometry (ICP-OES) which is a highly sensitive analytical tool for the detection of trace metals. The analyses were performed on samples of 1 μM free base porphyrin in aqueous solution kept in glass and quartz vials for 1 week at room temperature or under reflux for about 4 h. As references we performed ICP-OES analysis on water in glass vials at the two different temperatures and 1 μM aqueous solution of free base porphyrin in PMMA vials. Together with trace elements from water (Na, Be, Mg, Ca) and B and Si from the glass being present in all the samples, we detected the presence of Zn only in the samples of free base porphyrin kept in the glass/quartz vials. Quantitative analysis was performed and the presence of 5 μg L^–1^ of Zn for the sample kept at room temperature and 6 μg L^–1^ for the sample under reflux were detected. Zinc oxide is used in glass fabrication to reduce the coefficient of thermal expansion and to impart high brilliance and luster as well as high stability against deformation under stress [; http://www.zinc.org]. The high refractive index of zinc oxide makes it an excellent raw material for manufacturing optical glass [; http://www.ghchemicals.com]. When glass or silica is in contact with an aqueous solution of TPPS, the silanol groups exposed on the surface can temporarily bind the anionic sulfonato groups of the porphyrin through hydrogen bonds. This interaction brings the porphyrin core very close to the surface where zinc(ii) cations can be extracted and incorporated into the macrocyclic ligand. Temperature plays a role in metallation rates, in part due to favorable solvophobic interactions of the TPPS with the surface.

In order to explore the variability of the kinetics and chirality of the J-aggregates – and bearing in mind the potential adventitious presence of ZnTPPS in the solutions – we designed kinetic experiments by intentionally adding increasing amounts of the zinc derivative to freshly made aqueous solutions of TPPS stored in PMMA vials. Previous investigations have shown that when rather high concentration of porphyrin (10 μM) and hydrochloric acid (0.5 M) in a PF protocol are used to foster the J-aggregation process, no CD signals are detectable in solution.

**Fig. 3:**
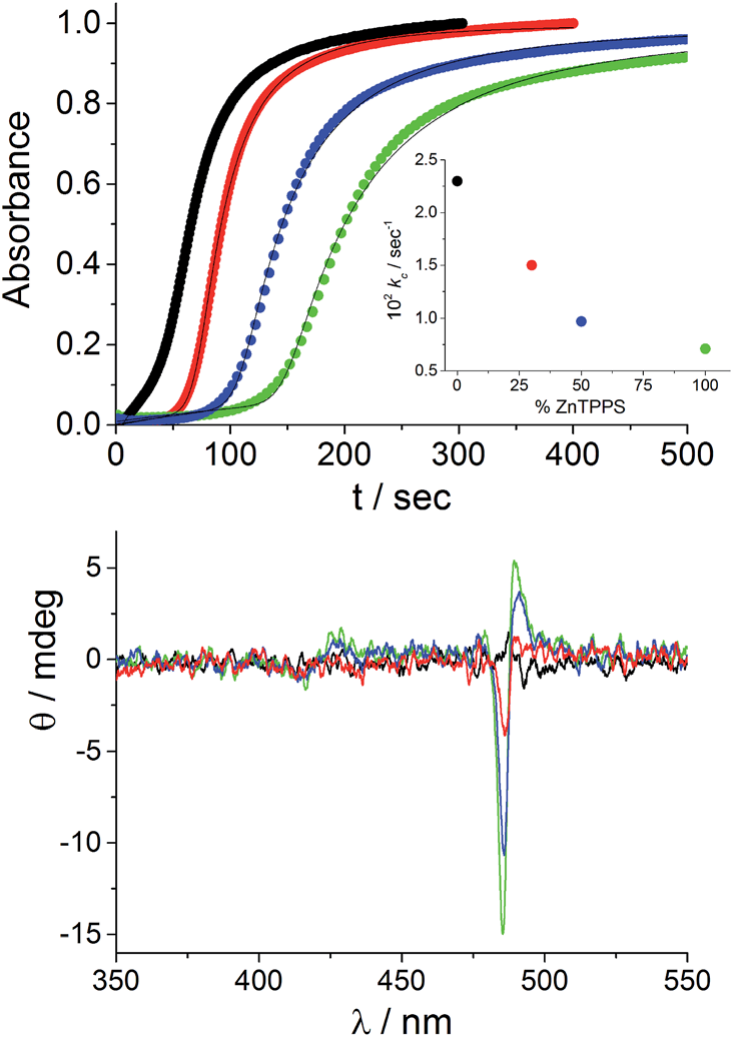
Extinction kinetic profiles for TPPS aggregation induced by HCl in the absence and in the presence of different amounts of ZnTPPS (upper plot) and the corresponding CD spectra (lower plot) at the end of the aggregation processes. The solid lines represent the best-fitting of the experimental data (*λ*_491 nm_) to eqn (1). In the inset the kinetic rate constants *k*_c_ are reported as a function of the percentage of added ZnTPPS concentration. Kinetic parameters are collected in Table 1. [TPPS]_0_ = 10 μM, [HCl] = 0.5 M; porphyrin solution stored in PMMA vials (black), ZnTPPS 30% (red), ZnTPPS 50% (blue), ZnTPPS 100% (green), *T* = 298 K, optical path length 2 mm.

We chose to begin our experiments using initial conditions that lead to a null CD spectrum as a “blank” experiment. When ZnTPPS is added to the starting porphyrin solution, the aggregation kinetics are markedly affected. The strongly acidic conditions induce an almost instantaneous demetallation of the porphyrin and the formation of the diacid species, which further self-arrange into J-aggregates. Fig. 3 shows the kinetic traces for the extinction taken at 491 nm corresponding to the time evolution of the J-aggregate in the presence of varying amounts of ZnTPPS added to the initial TPPS aqueous solution (the total porphyrin concentration is kept at 10 μM and ZnTPPS concentration is expressed as a percentage with respect to the total amount of porphyrin). All these kinetic profiles show the by now typical sigmoidal behavior, and the initial lag times increase with increasing amount of ZnTPPS. Analysis of the kinetic traces has been performed by the previously mentioned autocatalytic model,^23,24^ and the values of the parameters are collected in Table 1. The values of the rate constants for the uncatalyzed pathway, *k*_0_, and those for the catalyzed pathway, *k*_c_, decrease with increasing ZnTPPS, with the latter rate constant exhibiting a more pronounced inverse dependence on ZnTPPS concentration (Fig. 3, inset to upper panel). While *k*_0_ and *k*_c_ are in the range of the values reported in the literature for similar systems in aqueous solutions, *m* (∼4–8), the critical size for the nuclei, and *n* (∼6–16), the time exponent, are somewhat larger. CD spectra recorded at the end of the aggregation process are reported in Fig. 3 (lower panel). As expected, the CD signal is undetectable for the quite fast aggregation process in the freshly prepared aqueous solutions of neat TPPS in PMMA vials. On the contrary, CD spectra recorded at the end of the aggregation process for the samples containing ZnTPPS show a positive bisignate Cotton effect centered at the aggregate absorption bands.

**Table 1:** Kinetic parameters *k*_0_, *k*_c_, *m* and *n* for the aggregation of TPPS with HCl 0.5 M and the corresponding dissymmetry factor *g* at the end of the aggregation processes as a function of the percentage of added ZnTPPS^*a*^

% ZnTPPS	10^–4^ *k*_0_/s^–1^	10^–3^ *k*_c_/s^–1^	*m*	*n*	*g*
0	54 ± 1.5	23 ± 0.04	4.5 ± 0.1	6.4 ± 0.10	2 × 10^–5^
30	5.3 ± 0.6	15 ± 0.02	4.9 ± 0.1	10.1 ± 0.2	1.5 × 10^–4^
50	4.5 ± 0.9	9.7 ± 0.03	6.9 ± 0.5	13 ± 1	4.3 × 10^–4^
100	3.8 ± 0.9	7.1 ± 0.03	8.5 ± 0.8	16 ± 2	6.1 × 10^–4^

^*a*^PF protocol, total porphyrin concentration 10 μM, *λ* = 491 nm; *T* = 298 K. Data analysis according to eqn (1).

The sign and magnitude of the induced circular dichroism were further quantified by calculating the dissymmetry factor *g* (Δ*ε*/*ε*, Table 1). It is important to underline that *g* increases with increasing ZnTPPS concentration and, consequently, with decreasing aggregation rate. This behavior is in line with that observed on changing the TPPS concentration.^13^ Recalling that under the acidic conditions of these experiments the zinc(ii) cation is very rapidly removed from the porphyrin core,‡
‡Considering that the demetallation of ZnTPPS follows a rate law that is second order in [H^+^],^31^ we estimate a half-life time of 0.1 s for the demetallation of ZnTPPS in 0.5 M HCl (see also Fig. SI2†). our experimental evidence indicates that the zinc(ii) cation has a role during the nucleation step and the subsequent catalytic growth of the supramolecular assemblies. The direct involvement of ZnTPPS during the various steps of the assembly process is ruled out, since this species is readily converted into the diacid form of the TPPS porphyrin. An initial consideration is that, since ZnTPPS is less prone to self-aggregation than TPPS, the metal porphyrin provides a reservoir of TPPS in an initial monomeric form. Since the nucleation step is strongly controlled by the presence of small oligomers already formed in the TPPS stock solutions, the increased amounts of ZnTPPS lead to a better defined starting condition. Close inspection of the data in Table 1 shows that both *m* and *n* increase on increasing the amount of ZnTPPS added and, consequently, on increasing the concentration of free zinc(ii) cations (see the ESI, Fig. SI5†). This observation suggests that the metal ions are interacting with the TPPS units during the nucleation step modifying the number of monomers *m* required for the formation of the critical nucleus, which usually is in the range of 3–4.^13^ The parallel increase of the time exponent *n* with zinc(ii) concentration suggests that this cation is also playing a role in the subsequent catalytic growth of the nano-assemblies. Thus, our experimental findings offer a strategy for reducing the amount of initial preformed porphyrin oligomers which varies from sample to sample and which serve as nuclei for aggregation. We recommend that in studying acid-induced aggregation of TPPS, one begins by heating the porphyrin with an excess of Zn(ii). In our experiments we prepare a concentrated stock by heating a ten- to fifty-fold excess of ZnSO_4_ with TPPS in a boiling water bath for 20 minutes. The dilution of the resulting ZnTPPS stock for various kinetic/spectral experiments results in excess ZnSO_4_ concentrations of <0.1 mM.

**Fig. 4:**
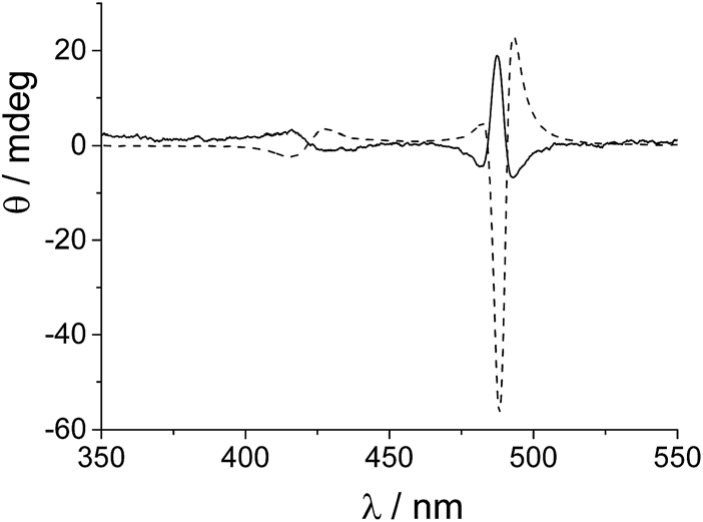
CD spectra for TPPS J-aggregates obtained in the absence (*g* = –5.8 × 10^–4^, solid line) and in the presence of ZnTPPS (*g* = 1.8 × 10^–2^, dashed line). [TPPS]_0_ = 7 μM, [HCl] = 0.5 M, ZnTPPS 5%, *T* = 298 K, optical path length 2 mm, *T* = 298 K.

A somewhat surprising result was obtained when aggregation was initiated with porphyrin aqueous solutions stored in PMMA vials in which no ZnTPPS is detectable in the fluorescence emission spectra. Over a restricted range of TPPS concentrations (5–8 μM, PF protocol), negative bisignate induced Cotton bands were observed. TPPS J-aggregates showing such negative exciton coupling have been reported in the literature.^16,17^ However, if very small amounts of ZnTPPS (<5%) are added to the starting porphyrin solutions under these experimental conditions, the final CD spectra show an inverted bisignate signal with respect to the aggregates obtained in the absence of Zn porphyrin (*i.e.*, a positive induced Cotton effect). Fig. 4 shows examples of CD spectra performed at the end of the aggregation process (porphyrin concentration 7 μM) with and without the addition of a small amount of ZnTPPS to the starting porphyrin aqueous solution.

**Fig. 5:**
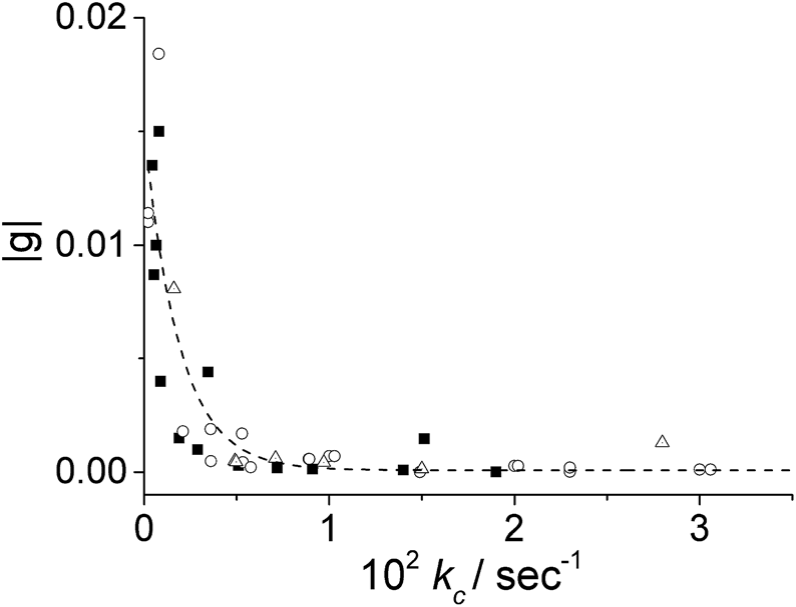
Plots of the dissymmetry *g*-factor of the resulting J-aggregates obtained under different experimental conditions as a function of *k*_c_ calculated by eqn (1). Aggregation was induced in porphyrin solutions stored in: PMMA vials (empty circles), glass vials (full squares), and in the presence of ZnTPPS (empty triangles). [TPPS] = 1–10 μM, [HCl] = 0.5 M, *T* = 298 K.

It is interesting to note that, apart from the inversion of the induced CD signal of the final aggregates, the factor *g* calculated for the J-aggregate obtained in the presence of ZnTPPS is more than one order of magnitude larger than that for the corresponding pure TPPS solution. A kinetic analysis of the extinction traces for the two assembling processes reveals that the value for the rate constant *k*_c_ is one order of magnitude smaller in the presence of ZnTPPS (5%). We expanded this approach by collecting and analysing kinetic data from experiments obtained using a different pre-treatment. In particular, we obtained data for samples at varying initial porphyrin concentrations stored in PMMA vials (empty circles) and in glass vials (full squares), and for aggregates induced in the presence of ZnTPPS (empty triangles). Fig. 5 shows that, whatever the pre-treatment of the sample, the absolute value of the dissymmetry factor *g* generally decreases on increasing the value of the kinetic rate constant *k*_c_. This experimental finding points out once again the importance of the aggregation rate on the induction of chirality in the supramolecular self-assembling process leading to TPPS J-aggregates. Also, it suggests a rationale for the discrepancies in the intensities of ICD signals reported in the literature.

## Conclusions

We have demonstrated that dilute aqueous solutions of TPPS under neutral conditions are able to extract zinc(ii) cations that are embedded in the surface of glass or silica where it is present as a factory additive. Upon ageing or increasing the temperature, metal insertion into the porphyrin core is accomplished. Even if the metal derivative is present in trace amounts – often not detectable with usual UV/Vis absorption techniques – it has a profound impact on the aggregation kinetics. This feature is very important for J-aggregates, where the aggregation rates closely correlate with the circular dichroism intensity of these nanostructures. Also the spectral differences between the parent free base and the zinc(ii) complex can lead to misinterpretation and incorrect assignments to the species present in the solution of this porphyrin. For example, recently, on the basis of the observed spectral changes and the partial lack of reproducibility, the binding of TPPS to carbohydrates has been reinterpreted as aggregation.^30^ We offer the present results as a possible basis for this disagreement in interpretation. These results should be heeded as a general warning for sulfonated and for other water soluble porphyrins that may be affected by the same or similar environmental problems. Furthermore, ZnTPPS could be regarded as a better model compound for mechanistic studies on the self-assembly process of the parent porphyrin under acidic conditions. The acid-induced demetallation and absence of any tendency towards self-aggregation exhibited by ZnTPPS lead to a very convenient way to generate the monomeric free porphyrin *in situ*. This approach avoids the problems related to ill-defined stock solutions where the presence of even small amounts of oligomers can markedly affect the kinetics, final structures and chirality of these supramolecular assemblies.

## Experimental

### Materials and methods

The porphyrin *meso*-tetrakis(4-sulfonatophenyl)porphyrin (TPPS) was purchased from Aldrich Co. as the tetrasodium salt. Zinc(ii) *meso*-tetrakis(4-sulfonatophenyl)porphyrin (ZnTPPS) was prepared according to literature methods, except where otherwise noted. Aqueous stock solutions of these porphyrins (200–500 μM) were prepared in dust-free Millipore water and stored in the dark in glass or PMMA vials. High purity HCl was purchased from Aldrich Co. or Fisher Scientific. Samples of J-aggregates were prepared rigorously following the porphyrin-first mixing protocol (PF).^13^ This consists of the addition of the appropriate volume of acid stock solution to a diluted porphyrin sample. Aggregation processes have been performed in 0.5 M HCl solution. UV/Vis spectra were collected using either a Jasco 550 spectrophotometer or a Hewlett-Packard mod. 8453 diode array spectrophotometer using 0.2–1 cm path length quartz cells. All glassware and cells used in the experiments were first cleaned with concentrated HNO_3_, H_2_SO_4_ or an acidic piranha mixture (H_2_O_2_/H_2_SO_4_ 1 : 3 v/v) followed by standard ionic detergents, and eventually rinsed with deionized and HPLC grade water. Kinetic experiments were carried out in the thermostated compartment of the spectrophotometer, with a temperature accuracy of ±0.1 K. The analyses of the kinetic profiles have been performed by a non-linear fit of the extinction data according to the following equation:

1 Ext = Ext_inf_ + (Ext_0_ – Ext_inf_)(1 + (*m* – 1){*k*_0_*t* + (*n* + 1)^–1^(*k*_c_*t*)^*n*+1^})^–1/(*m*–1)^

where Ext_0_, Ext_inf_, *k*_0_, *k*_c_, *m* and *n* are the parameters to be optimized (Ext, Ext_0_ and Ext_inf_ are the extinctions at time *t*, at starting time and at the end of aggregation, respectively).

The circular dichroism (CD) spectra were recorded on an Aviv Model 410 or a JASCO J-720 spectropolarimeter, equipped with a 450 W xenon lamp. The ellipticity was obtained by calibrating the instruments with a 0.06% aqueous solution of *R*-camphorsulfonic acid. CD spectra were corrected for both the cell and solvent contributions. To quantify the sign and magnitude of the supramolecular chirality we used the dissymmetry factor *g* = Δ*ε*/*ε* = ΔExt/Ext, with ΔExt = *Θ*/32 980, where ΔExt is in extinction units and *Θ* is the ellipticity in mdeg.

Fluorescence emission spectra were performed on a Jasco FP-750 spectrofluorimeter. Time resolved fluorescence emission measurements were performed on a Jobin Yvon-Spex Fluoromax 4 spectrofluorimeter using the time-correlated single-photon counting technique. A NanoLED (*λ* = 390 nm) has been used as the excitation source. ICP-OES analysis was carried out by atomic emission spectroscopy with ICP Horiba Jobin Yvon ULTIMA 2 having a radial configuration. The monochromator was a 1 m Czerny–Turner JY 1000 S-type having a holographic grating with 2400 grooves per mm. The operating conditions and instrumental parameters for ICP-OES analyses are reported in the ESI (Tables SI1–2†). The linearity of the instrumental response for the particular element has been checked through standard solutions, prepared at different concentration levels using appropriately diluted solutions obtained from standard solution in 2% HNO_3_. The analysis of each solution was repeated three times. The calibration line obtained showed excellent linearity over the concentration ranges investigated, with a linear regression coefficient *R* ^2^ ≥ 0.999. The detection limit (LOD) 0.9 μg L^–1^ has been determined as 3.3*σ*/*S*, and the quantification limit (LOQ) 2.97 μg L^–1^ was calculated as 10*σ*/*S*, where *σ* and *S* are the residual standard deviation and the slope of the regression line, respectively.
