# Supplementary material for: Effect of zinc cations on the kinetics of supramolecular assembly and the chirality of porphyrin J-aggregates
Source: Chem Sci. 2016 Sep 29;8(2):961–7. doi: 10.1039/c6sc02686a (PMC5354048; doi:10.1039/c6sc02686a)
Supplement: Supplementary file 1 [file SC-008-C6SC02686A-s001.pdf]

Electronic Supplementary Material (ESI) for Chemical Science.
This journal is © The Royal Society of Chemistry 2016

Effect of zinc cations on kinetics and chirality in porphyrin J-aggregates

Andrea Romeo,[a,b] Maria Angela Castriciano,[a]* Roberto Zagami,[a] GianMichele Pollicino,[c] Luigi Monsù Scolaro[a,b] * and Robert F. Pasternack[d]

[a] Istituto per lo Studio dei Materiali Nanostrutturati ISMN-CNR c/o Dipartimento di Scienze Chimiche, Biologiche, Farmaceutiche ed Ambientali, University of Messina V.le F. Stagno D'Alcontres, 31 98166 Messina, Italy

[b] Dipartimento di Scienze Chimiche, Biologiche, Farmaceutiche ed Ambientali, University of Messina and C.I.R.C.M.S.B V.le F. Stagno D'Alcontres, 31 98166 Messina, Italy.

[c] Dipartimento di Scienze Biomediche, Odontoiatriche e delle Immagini Morfologiche e Funzionali, Sezione SASTAS, University of Messina, Messina, Italy.

[d] Department of Chemistry and Biochemistry, Swarthmore College, Swarthmore, Pennsylvania PA 19081, USA.

Supporting Information

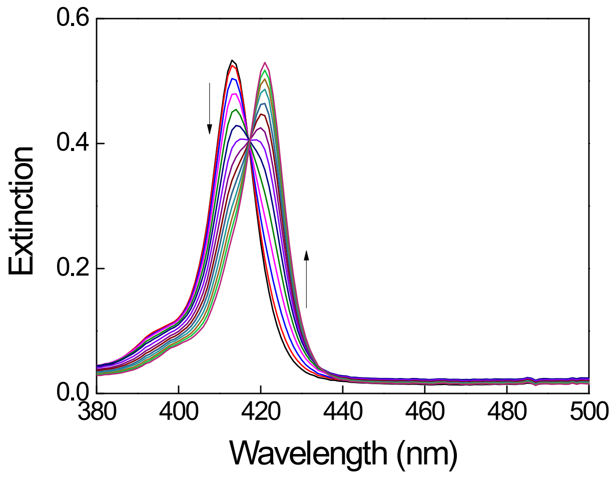

Figure SI1 UV-vis spectral changes during *TPPS* aqueous solution thermal annealing in glass. Decreasing of 414 nm specie and increasing of 422 nm specie. (scanning time 5400 s). [TPPS] = 1 μM; T = 330 K.

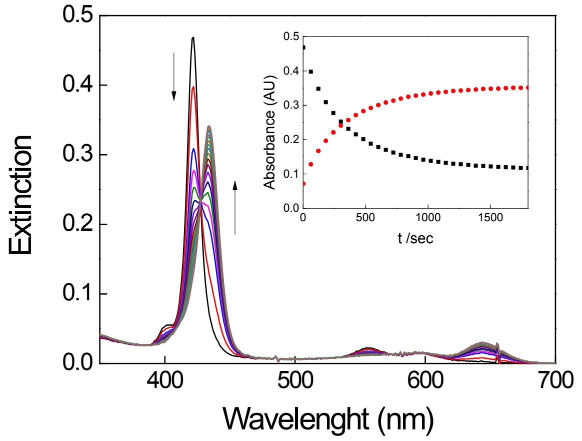

Figure SI2 UV-vis spectral changes for Zn*TPPS* demetallation (scanning time 60 s). In the inset the corresponding UV-vis kinetic profile λ = 422 nm (black) and λ = 434 nm (red). [TPPS] = 1 μM, [HCl] = 0.01 M, T = 298 K.

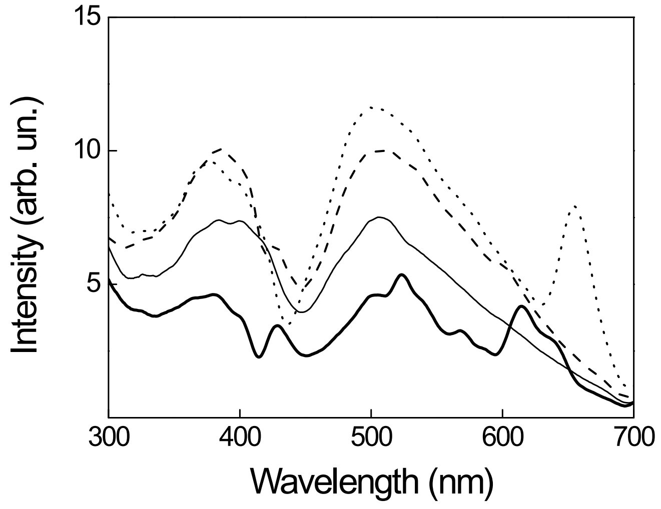

Figure SI3. RLS spectra of water (black thin line), TPPS freshly made aqueous solution (full thick line), after thermal annealing (dashed line) and soon after acidification ([HCl] = 0.5 M) of the thermal annealed solution (dotted line). [TPPS] = 1 μM; T = 298 K.

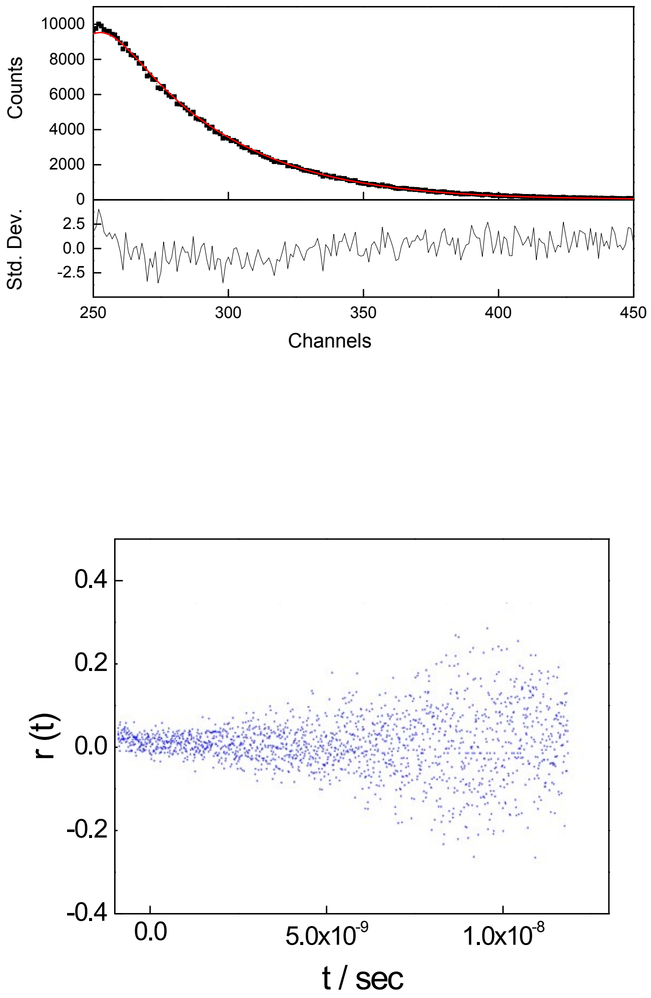

Figure SI4. Fluorescence emission decay (upper) and time resolved fluorescence anisotropy (lower) of TPPS after thermal annealing, T = 298 K, $\lambda_{ex.}$ = 390 nm, $\lambda_{em.}$ = 606nm.

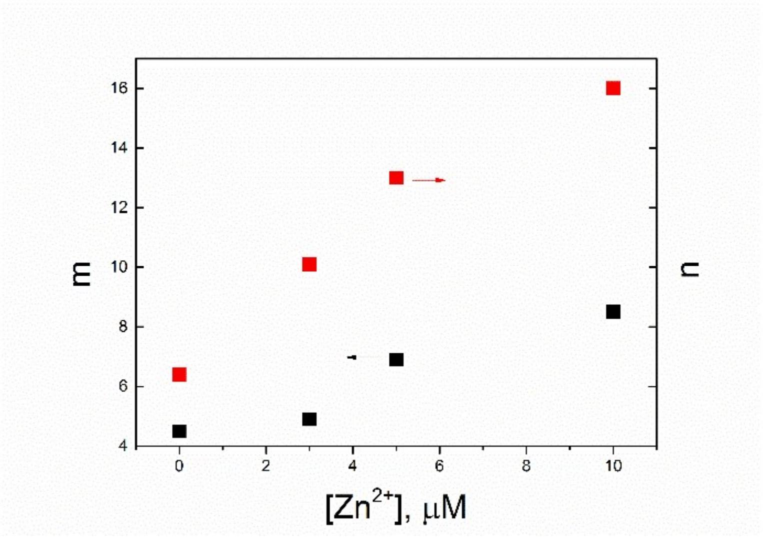

Figure SI5. . Kinetic parameters m and n for the aggregation of TPPS with HCl 0.5 M as function of concentration of Zn(II) in solution. Data from table 1.

Table SI1. Conditions for ICP-OES analysis.

Parameters	
Power generator	1000 W
Plasma gas flow	12 L min^{-1}
Gas flow support	0.2 L min^{-1}
Nebulising gas flow	1 L min^{-1}
Nebulising pressure	2.98 bar
Speed peristaltic pump	20 rpm
Flow sample introduction	0.99 mL min^{-1}

Table SI2. Acquisition parameters for ICP-OES analysis.

Element	*λ (nm)*	*Slits (μm)*	*Acquisition mode*	*Integration time (sec)*
Zn	213.856	20x15	Max	4
